# Effect of one comprehensive education course to lower anxiety and depression among Chinese breast cancer patients during the postoperative radiotherapy period - one randomized clinical trial

**DOI:** 10.1186/s13014-018-1054-6

**Published:** 2018-06-14

**Authors:** Zhensheng Li, Wenhui Geng, Junpu Yin, Jun Zhang

**Affiliations:** Department of Radiation Oncology, the Fourth Hospital of Hebei Medical University, 169 Tianshan Street, Shijiazhuang, 050035 China

**Keywords:** Breast cancer, Postoperative radiotherapy, Anxiety, Depression, Randomized controlled trial

## Abstract

**Background:**

We investigated the effectiveness of one education course to lower the severity of anxiety and depression symptoms among breast cancer (BC) patients during radiotherapy (RT).

**Methods:**

All 290 one-sided BC patients were evenly randomized into intervention or control arm. “Intervention” patient was additionally provided with one three-hour course on psychological stresses and management skills. Changes of anxiety and depression score and their 3-level severity category (‘normal’, ‘borderline’ and ‘abnormal’ scored 0–7, 8–10 and 11–21, respectively) from HADS questionnaire over RT were evaluated by multivariable linear and ordinal logistic regressions.

**Results:**

Response rates were 94 and 100% by “intervention” and “control” arm, respectively. Means of score changes by “intervention” and “control” (*n* = 145) were + 0.59 (SD = 2.47) and + 0.11 (SD = 2.55) for anxiety and + 0.81 (SD = 2.81) and + 0.45 (SD = 2.77) for depression scores, respectively. ‘Abnormal’ anxiety and depression patients were 4.1 and 6.9% at baseline and 4.8 and 6.9% at end of RT at ‘control’ arm; those rates were 6.6 and 7.4%, and 8.8 and 10.3% at ‘intervention’ arm, respectively. Both changes on anxiety and depression measurements between two arms were all insignificant (*p > 0.20*).

**Conclusions:**

One education course did not reduce the score and severity of anxiety and depression symptoms over RT period.

**Trial registration:**

Chinese Clinical Trial Registry #: ChiCTR-IIR-16008818 at www.chictr.org.cn.

## Background

Anxiety and depression are commonly observed among breast cancer (BC) patients over any period of cancer screening, breast lump imaging, cancer diagnosis, treatments, and tumor recurrence, and surveillance follow-up [1–7]. Especially for BC patients who are experiencing the intensive treatments, anxiety and depression contribute various signs and symptoms which often indicate or heighten the pain expectancy, sleep disturbances, gastrointestinal symptoms, poor compliance with medications, and thus further complicate BC management [8]. As frequently observed, many studies have demonstrated that anxiety and depression at any level could substantially interfere with the quality of life (QoL) of patient and possibly his/her survival if not evaluated and treated timely [2, 9, 10].

The exact etiological mechanism of BC associated anxiety and depression is still unclear. However, many studies have found that both medical and non-medical factors are deeply involved in the emergence of anxiety and depression [1, 11]. Young age, female, black and Asia races, limited cancer knowledge, active treatments, and the life-changing complications as results of therapies have been identified as the contributing factors [4, 7, 12, 13]. More specifically, BC surgery (either lumpectomy or mastectomy) and other treatments including chemotherapy (CTx), radiotherapy (RT), and endocrine therapy (ET), are found to be the most significant elements in exacerbating these psychological symptoms [14–17]. There are many other factors also involved such as marital status (either single or divorced), low social status (like education and income), lack of insurance, and unemployment [18, 19]. Although there are reportedly as high as 40–80% prevalent rates of anxiety and depression symptoms, only 3–5% of BC patients have experienced psychosocial distress serious enough to meet the disorder diagnosis under the fourth revised edition of the Diagnostic and Statistical Manual of Mental Disorders (DSM-IV-TR) at any time point forward after BC diagnosis [20, 21]. Nonetheless, given the high prevalence of anxiety and depression, BC patients especially those who undergo the series of therapy modalities, are strongly recommended by guidelines to undergo regular, psychosocial assessments with professionals like psychiatrist, psychologist, or pastoral counselors especially over the subsequent CTx and RT episodes after the surgery [22, 23]. Therefore, how to minimize the impact of anxiety and depression symptoms on QoL of BC patients systemically has become one challenging but essential part of BC integrated medical care [24–26]. The timely prevention and intervention are clinically required.

Similar to the management on chronic diseases like diabetes mellitus and hypertension, education regarding BC as a form of non-pharmacologic interventions has been found to be associated with higher patient compliance of ET, regular exercise, and healthy and well-balanced diets [27, 28]. Some clinical studies have also shown that the early educational or psychological interventions could lower the incidence and severity of anxiety and depression symptoms and improve their survival [29–32]. Similarly to other countries, the necessary postoperative RT and ET in China are administered after the surgery and 6–8 cycles of CTx. With fear to RT and side effects of CTx and ET, many BC patients at this stage have experienced the anxiety and depression symptoms of poor appetite, nervousness, panic, sadness, tiredness, apathy, sleeping disorder, feelings of worthlessness and helplessness, and even suicidal tendency [17, 33–35]. At this stage, the adequate education on RT knowledge including high energy x ray from accelerator, new technique of using three-dimensional conformal radiotherapy (3DCRT) or intensity-modulated radiotherapy (IMRT) with good control of dosage on normal organ at risk was found by some studies to be able to reduce some fears and anxious feelings [36–38]. Therefore, one overdue comprehensive education course on BC and its managements before RT could be theoretically effective. Given these findings, we designed one randomized clinical trial (RCT) to test whether educational intervention prior to RT can impact the anxiety and depression symptoms presented among RT eligible BC patients.

The objective of this trial was to determine if a comprehensive education course on BC disease knowledge and its management can significantly reduce the incidence and/or severity of anxiety and depression symptoms measured through The Hospital Anxiety and Depression Scale (HADS) during the RT period among Chinese BC patients.

## Methods

### Study design and participants

This study was a two-arm, one centre, randomized, phase 3 trial comparing the effectiveness of one education course versus none impacting the anxiety and depressoin symptom severities among BC patients during RT. All BC patients admitted for postoperative RT at the Fourth Hospital of Hebei Medical University from Nov. 2013 to Feb. 2015 were screened for this trial enrollment by two study coordinators with handouts. The inclusion criteria included female, age ≥ 18 years old, one-sided BC diagnosis, the modified radical mastectomy (MRM) or breast conserving surgery (BCS) performed within 6 months, ECOG performance score 0 or 1, and signed Informed Consent Form (ICF). The exclusion criteria included recurrence, pregnant women, dementia, the history of alcohol or narcotics abuse, current or past diagnosis or treatment of psychological or psychiatric disorders, current use of any Chinese herbs, and inability to understand the HADS questionnaires even with oral help from family members. The study was approved by the Medical Ethics Committee of the Fourth Hospital of Hebei Medical University. Under the trial proposal, two hundred and ninety BC patients were enrolled into this study.

### Randomization and intervention

Each enrolled patient was randomly allocated (in a 1:1 ratio) into either “intervention” (I) or “control” (C) arms immediately after the ICF signature was obtained and by using a randomization schedule. The schedule was generated from using SAS procedure Proc PLAN with block size 4. Masking was not used in this trial. The patient, study coordinator, researcher and others were aware of the assigned procedures. Under the standard medical care guideline of the hospital, all patients were offered a15-minute educational course on basic knowledge of BC and RT on the admission day. For “I” patient only, one comprehensive education course designed for this trial was scheduled at one meeting room. The content of the three-hour course was formulated by the study team in advance and was delivered in slides and question/answer format twice weekly by two well-trained physician researchers prior to the patient’s first RT session. Only one course was allowed for each patient to attend during the entire study period. In brief, the entire course covered the BC basics (breast anatomy, cancer development, diagnosis, staging, treatment, and survivorship) in plain language with emphasis on BC-related psychiatry and psychology management. It also provided many didactic presentations such as psychosocial and nutrition intervention skills. In short, the interventional tools included the timely consultation with the primary care physician, group-talking techniques and healthy lifestyle maintenance (healthy diet, sufficient sleep, and regular exercise) in order to reduce BC-related stress symptoms. Because of individual reasons, a few “I” arm patients did not attend the course after one attempt of reminders and one course rescheduling failed.

### Trial size calculation

The HADS has been widely used to measure distress in BC patients [39–41]. Its Chinese version has also been validated and used among Chinese BC patients in several studies with good reliability estimates of Cronbach’s α over 0.80 for anxiety and depression subscale [42–44]. The hypothesis of this trial was that one education course would reduce the mean of anxiety or depression score measured by HADS to be less than 0.5 (defined as the minimal detectable and clinical meaningful difference). Under a randomized two-arm parallel trial design, a total 242 patients would be required to detect a intervention difference at a two-side *α* = 0.01 significant level with *β* = 90% probability power.

### Demographic and clinical data at baseline

All demographic and key clinical data were collected through the pre-defined case report form at baseline which was defined as the signed date of ICF. Data included age, height, weight, marital status, highest level of education, self-reported income level, menopause status, BC side, TNM stage, and surgery type (MRM vs. BCS). Body mass index (BMI) was calculated from weight (kg) divided by squared height in meters.

### The hospital anxiety and depression scale measure and delivery

All patients were asked to fill in the HAD questionnaire within ±1 day of the first and last RT date. The first measurement was mandatory and reported as the baseline score. In literature, both English and Chinese versions of the HAD questionnaire have been validated in many studies for cancer patients [41, 44]. In brief, it consists of 14 items and contributes to two subscales of anxiety (HADS-A) and depression (HADS-D) domains. Both HADS-A and HADS-D scores are calculated from the sum of 7 different item scores in a four-point Likert scale 0 to 3. A high score of HADS-A or HADS-D indicates high levels of anxiety and depression severity, respectively. To categorize the measures, three levels of ‘normal’, ‘borderline’ and ‘abnormal’ are classified by the score range of 0–7, 8–10 and 11–21 for each subscale [45]. In this study, both continuous and categorical HADS-A and HADS-D measured changes were analyzed. Both HADS-A and HADS-D items presented high internal consistency with the calculated Cronbach’s α coefficient range 0.80–0.85. In addition, the binary ‘abnormal’ status change of patient at each subscale was analyzed as well. Please note that the score changes of the HADS-A and HADS-D subscales were defined as the score at the end of RT minus the baseline one.

Per the protocol, all patients filled the HADS questionnaire on site herself with one written instruction available while one research staff and one immediate family member (optional) could be there for assistance if needed [39]. All “I” arm patients attended the education course in 0–5 days after the HADS questionnaire was filled. The patient was not allowed to access the baseline records of HADS at the second time of filling.

### Radiotherapy and hormonal treatment

For each patient, RT was delivered through 3DCRT or IMRT with 6-MV protons of accelerator at the discretion of individual treating physicians. All patients completed the planned RT therapy in doses of 50Gy/25 fractions/5–6 weeks for the chest and supraclavicular lymph node regions for MRM patients, and 50Gy/25 fractions/5–6 weeks for the whole breast plus the tumor bed with β rays boost 10 Gy/5 fractions/1 week for BCS patients. Under the study protocol, less than 5 consecutive day of RT interruption was allowed in case of treating radiation dermatitis, lymphatic edema, and bone marrow suppression. For all patients with positive ER or PR (defined as > 2% cells), the hormonal treatments of taking mainly Tamoxifen or Letrozol were provided since the admission. No chemotherapy was used during the RT sessions.

### Statistical analysis

Descriptive statistics were used to characterize the patient demographic and clinical data collected at baseline. To compare means or percents between two groups, student’s t-test and Chi-square test (the exact test if applicable) were used, respectively. Simple ANOVA was applied for mean comparisons among more than two groups. To account for the confounding effects of covariates on means or multinomial level percents, the multivariable linear regression (MLR) and multinomial ordered logistic regression (MOLR) models were used for referential analyses. Age, surgery type (MRM vs. BCS), TNM stage, education level, and self-rated income group were considered stratifying factors or covariates in these analyses. HT treatment status was finally excluded from the covariate list given its consistent insignificant effects found in all statistical models. For presentation, the estimated coefficient and their associated *p* value were cited from the univariate linear regression and MLR models. From the MOLR models, the Odd Ratio (OR) and its 95% confidence interval (CI) were presented. All statistical analyses were conducted using SAS v9.2 (SAS Institute, Cary, NC). The significance level was two-sided *p* ≤ 0.05.

## Results

### Participants and analysis population

Overall, 290 patients who met the study inclusion and exclusion criteria and signed the ICF were randomized into the study. Each arm enrolled 145 patients. However, nine (6.2%) of 145 “I” patients were excluded from the analysis because they did not attend the education course at all. Per the protocol, the analysis population was defined as the randomized patients who actually attended the education course, and filled at least one item of the HADS at baseline. No score imputations for the missing HADS items were conducted given that only two (< 1%) patients in the analysis population did not respond 1–2 items either at baseline or after RT due to the apparent negligence. Given the nature of this study, the intention-to-treatment (ITT) population was not chosen for analysis.

Table 1 shows the balanced demographic and clinical characteristics of the analysis population overall and by study arm. Among the total 281 patients in the analyzed population, the means were 46.2 years old for age and 26.8 kg/m^2 for BMI. The majority (97.5%) had MRM and 51.6% were diagnosed with left-sided BC. In terms of pathological TNM stage, 19 (6.8%) patients were ‘undetermined’ because their primary tumor size exact records could not be found from charts, and 51.6% (145/281) were classified as stage II, and 29.9% (84/281) as stage III. By self-reported income level, 25.6% patients claimed themselves to be ‘poor’ and no one reported having ‘high’ income. No statistical differences between the two arms on demographic and clinical data were found (Table 1). Because only one patient reported as ‘single’, the marriage status (data not shown) was not analyzed.

**Table 1:** Baseline characteristics of study analysis population

Variable		Control Arm		Intervention Arm		All		*p* ^b^
			(%)		(%)		(% or range)	value
Age (years old)	n	145		136		281		ns
	mean ± std.^a^	47.3 ± 8.80		45.1 ± 8.49		46.2 ± 8.70		
	median	48		46		47	(25–67)	
Height (cm)	n	145		136		281		ns
	mean ± std	158.4 ± 4.87		158.1 ± 4.32		158.2 ± 4.61		
	median	158		158.5		158.5	(139–172)	
Weight (kg)	n	145		136		281		ns
	mean ± std	67.4 ± 9.77		66.8 ± 8.81		67.1 ± 9.30		
	median	66.0		66.5		66.0	(45–103)	
BMI (kg/m^2)	n	145		136		281		ns
	mean ± std	26.9 ± 3.58		26.7 ± 3.37		26.8 ± 3.48		
	median	26.2		26.6		26.4	(18.2–39.0)	
Education	no school	0	(0.0)	2	(1.5)	2	(0.7)	ns
	elementary	13	(9.0)	18	(13.2)	31	(11.0)	
	middle school	70	(48.3)	74	(54.4)	144	(51.3)	
	high school	32	(22.1)	25	(18.4)	57	(20.3)	
	college & up	30	(20.7)	17	(12.5)	47	(16.7)	
Income level	poor	33	(22.8)	39	(28.7)	72	(25.6)	ns
	low	62	(42.8)	60	(44.1)	122	(43.4)	
	middle	50	(34.5)	37	(27.2)	87	(31.0)	
Tumor side	left	71	(49.0)	74	(54.4)	145	(51.6)	
	right	74	(51.0)	62	(45.6)	136	(48.4)	
Surgery	MRM^a^	143	(98.6)	131	(96.3)	274	(97.5)	
	BCS^a^	2	(1.4)	5	(3.7)	7	(2.5)	
TNM Stage	undetermined	10	(6.9)	9	(6.6)	19	(6.8)	ns
	I	15	(10.3)	15	(11.0)	30	(10.7)	
	II	78	(53.8)	67	(49.3)	145	(51.6)	
	III	40	(27.6)	44	(32.4)	84	(29.9)	
	IV	2	(1.4)	1	(0.7)	3	(1.1)	

^a^*std* standard deviation, *BMI* body mass index, *MRM* modified radical mastectomy, *BCS* breast conserving surgery

^b^*p* value was from the Student’s t test or simple Chi-square or exact test. *ns* not significant at *p* > 0.20

### Anxiety and depression score and severity

At baseline and completion of RT (i.e. after RT), both anxiety and depression scores did not show significant differences between the two arms (Table 2). However, the anxiety score was significantly increased from its baseline mean 5.15 to 5.49 after RT (*p* = 0.02, t-test) but with the same median 5.0 (*p* > 0.05, median test). The depression score also increased significantly from its baseline mean (median) 4.79 (4.0) to 5.40 (5.0) after RT (both t and median test *p* values = 0.00). Among the score-based severity levels, the ‘abnormal’ anxiety percent increased from baseline 5.3% (15/279) to the RT end 6.8% (19/279). And the ‘abnormal’ depression percent also increased from baseline 7.1% (20/218) at baseline to the RT end 8.5% (24/201). However, the simple independent proportion comparison tests did not show any difference of the ‘abnormal’ rates of both anxiety and depression between baseline and ‘after RT’. To accurately appreciate the differences of their changes between the two study arms, both linear and ordered logistic regression model analyses were applied.

**Table 2:** Anxiety and depression score and severity by study arm

Measure	Arm	Baseline			After RT			*p*-value^a^
		n	mean ± std	median	n	mean ± std	median	
Anxiety	Control	144	5.03 ± 3.17	5.0	144	5.13 ± 3.15	5.0	
	Intervention	135	5.29 ± 3.27	5.0	135	5.87 ± 3.18	6.0	
	all	279	5.15 ± 3.22	5.0	279	5.49 ± 3.18	5.0	0.02
Depression	Control	145	4.57 ± 3.46	4.0	143	5.01 ± 3.65	5.0	
	Intervention	136	5.02 ± 3.41	5.0	135	5.81 ± 3.68	5.0	
	all	281	4.79 ± 3.44	4.0	278	5.40 ± 3.68	5.0	0.00
		normal	borderline	abnormal	normal	borderline	abnormal	
		n (%)	n (%)	n (%)	n (%)	n (%)	n (%)	
Anxiety	Control	113(77.9)	25(17.2)	6(4.1)	112(77.2)	25(17.2)	7(4.8)	
	Intervention	109(80.2)	17(12.5)	9(6.6)	100(73.5)	23(16.9)	12(8.8)	
	all	222(79.0)	42(15.0)	15(5.3)	212(75.4)	48(17.1)	19(6.8)	ns
Depression	Control	117(80.7)	18(12.4)	10(6.9)	110(75.9)	23(15.9)	10(6.9)	
	Intervention	101(74.3)	25(18.4)	10(7.4)	91(66.9)	30(22.1)	14(10.3)	
	all	218(77.6)	43(15.3)	20(7.1)	201(71.5)	53(18.9)	24(8.5)	ns

*RT* radiotherapy, *std* standard deviation, *ns* not significant at *p* > 0.20

^a^*p* value was from the simple Student’s t-test or proportions equal test in comparing the overall mean or ‘abnormal’ category percents before and after radiotherapy for anxiety and depression measures. Patients with missing score were treated as one category for percent calculation but not reported

### Changes of anxiety and depression score over RT

Table 3 presents the comparisons of the anxiety and depression score changes between two study arms and by other demographic and disease character groups at baseline. Overall, the anxiety and depression score changes were significantly positive with overall means of + 0.34 and + 0.62 respectively (both paired t-test *p* < 0.05, comparing with zero). Their 95%CIs of the mean differences of anxiety and depression were estimated to be 0.05–0.64 and 0.29–0.95, respectively. However, the insignificant median test on the anxiety (*p* = 0.14, test if the population median values equal 0) did not support its score increase. The median test supported the significant depression score increase over RT.

**Table 3:** Anxiety and depression score change by patient characters of analysis population^a^

			Anxiety Change		*p-*value^a,b,c^		Depression Change		*p-*value^a,b,c^
		n	Mean ± std	MD		n	Mean ± std	MD	
All		277	0.34 ± 2.52	0.0	0.02	278	0.62 ± 2.79	0.0	0.00
Arm	Control	143	0.11 ± 2.55	0.0	ns	143	0.45 ± 2.77	0.0	ns
	Intervention	134	0.59 ± 2.47	1.0		135	0.81 ± 2.81	0.0	
Age	< 40	64	0.50 ± 2.25	0.0	ns	65	0.57 ± 2.40	0.0	ns
	41–50	120	0.52 ± 2.52	0.0		120	0.71 ± 2.51	0.0	
	> = 50	93	0.01 ± 2.68	0.0		93	0.55 ± 3.35	0.0	
BMI (kg/m^2)	< 23.9	55	−0.24 ± 1.90	0.0	ns	54	0.35 ± 2.27	0.0	ns
	24.0–27.9	125	0.58 ± 2.77	0.0		127	0.61 ± 3.03	0.0	
	> = 28.0	97	0.37 ± 2.45	0.0		97	0.79 ± 2.74	0.0	
TNM Stage	undetermined	19	−0.58 ± 3.06	−1.0	ns	19	−0.47 ± 1.93	−1.0	ns
	I	30	0.67 ± 2.77	0.5		30	0.83 ± 3.76	0.0	
	II	143	0.21 ± 2.41	0.0		144	0.40 ± 2.51	0.0	
	III	82	0.65 ± 2.48	1.0		82	1.23 ± 2.93	1.0	
	IV	3	1.00 ± 1.00	1.0		3	−0.33 ± 2.52	0.0	
Education	no school	2	5.00 ± 1.41	5.0	0.01	2	1.50 ± 0.71	1.5	ns
	elementary	31	0.97 ± 3.14	1.0		31	1.55 ± 3.69	2.0	
	middle school	142	0.54 ± 2.49	0.0		143	0.71 ± 2.85	0.0	
	high school	55	−0.56 ± 2.51	0.0		55	−0.02 ± 2.78	0.0	
	college & up	47	0.19 ± 1.68	0.0		47	0.47 ± 1.67	0.0	
Income	Poor	71	0.80 ± 2.61	0.0	0.06	72	1.11 ± 3.09	1.0	ns
	Low	121	−0.11 ± 2.59	0.0		121	0.26 ± 2.46	0.0	
	Middle	85	0.60 ± 2.24	0.0		85	0.73 ± 2.92	0.0	
Tumor	Left	141	0.52 ± 2.51	0.0	ns	142	0.83 ± 2.88	0.0	ns
	Right	136	0.15 ± 2.52	0.0		136	0.40 ± 2.68	0.0	
Surgery	MRM	270	0.31 ± 2.49	0.0	ns	271	0.58 ± 2.72	0.0	ns
	BCS	7	1.57 ± 3.36	1.0		7	2.14 ± 4.78	0.0	

^a^score change means the score after radiotherapy minus the baseline score. *Std* standard deviation, *MD* median, *MRM* modified radical mastectomy, *BCS* breast conserving surgery, *ns* not significant (*p* > 0.10)

^a^For analyses at single level among ‘All’ patients, the paired t test *p* values are shown. However, *p* values of the median test (if equal zero) are 0.14 and 0.04 (not shown in table) for the anxiety and depression score change, respectively

^b^for analyses stratified by 2 study arms, *p* values from ANOVA are presented; meanwhile, *p* values from the non-parametric Kruskal-Wallis test are 0.09 and 0.45 (not shown in table either) for anxiety and depression score change, respectively

^c^for other analyses not stated above, *p* values from the Chi-square or Fisher exact test (if applied) are presented

Through simple ANOVA analyses, Table 3 indicated that there were no significant differences of anxiety or depression score changes over RT between or among the two arms, age group, TNM stage, tumor side and surgery type with all *p* > 0.10. However, low education and poor income levels were found to associate with high increase of anxiety score at significant (*p* = 0.01) and marginally significant levels (*p* = 0.06), respectively. Regression analyses were subsequently conducted to compare the score change of anxiety and depression between the two arms. Table 4 presented the results of univariate and MLR analyses which showed there were no significant differences of both changes between the “I” and “C” arms (all *p* values > 0.10). While the education level was found to be negatively and significantly associated with high anxiety increase at univariate and MLR models (both p = 0.01, data not shown), none of the other covariates contributed significantly to either anxiety or depression score changes (data not presented in Table 4). Surprisingly, the BCS patients tended to have marginally statistical larger increase scores of anxiety and depression changes (both *p* = 0.06) at MLR models only (data not showed in Table 4 either).

**Table 4:** Linear regression analysis on anxiety and depression score changes of analysis population^a, b^

Dependent Variable	Arm	Univariate Analysis				Multivariable Analysis			
		EST.	SE	T	*P* value	EST.	SE	T	*P* value
Anxiety change	Int.	0.478	0.302	1.58	0.11	0.102	0.309	0.33	0.74
Depression change	Int.	0.360	0.335	1.08	0.28	0.206	0.359	0.57	0.57

*EST* coefficient estimate, *SE* standard error, *T* t value, *Int.* intervention (vs. control)

^a^A few patients were excluded from the univariate and multivariate modeling because of their absence of anxiety or depression score changes or their TNM stage missing status. Covariates at multivariable regression analysis include age, education, BMI, TNM stage, income, breast tumor side, and surgery type

^b^Reference groups at linear regression analyses are the ‘control’ group of study arm, ‘Stage IV’ of Stage, ‘college and up’ of education level, ‘right-sided’ of tumor side, and ‘BCS’ of surgery type where BCS means the breast conserving surgery

### Changes of anxiety and depression level over RT

To understand how anxiety and depression severity levels changed differently between ‘I’ and ‘C’ arms, both basic and multivariable MOLR models were conducted. Please note that these analyses should be considered to be supplemental to the score change analyses above and would be more meaningful in clinical aspects (Table 5). That is to say, the three levels of anxiety and depression scores - ‘normal’, ‘borderline’ and ‘abnormal’ - were collectively analyzed in order to reduce the possible significant impact of individual score assessment sensitivity variations on analysis result. Table 2 (low part) showed that the percents of ‘normal’, ‘borderline’ and ‘abnormal’ anxiety scores at baseline were 79.0%, 15.0 and 5.3%, respectively. At the end of RT, these percents were 75.4, 17.1 and 6.8%, respectively. In consideration of depression severity level, these percents were 77.6, 15.3 and 7.1% at baseline, and 71.5, 18.9 and 8.5% at end of RT. The same covariates at MLR models were utilized at MOLR analyses. The basic MOLR model had the independent variables of the trial arm and levels of score-based anxiety or depression severity only. Table 5 demonstrated that the baseline anxiety and depression severity level significantly contributed to their corresponding levels at end of RT (all *p* values < 0.001) in all models. With regard to anxiety or depression levels, ‘I’ patients had no statistical differences of their changes over RT, compared to the ‘C’ patients. However, the adjusted OR (95%CI, *p* value) from multivariable MOLR models for anxiety was calculated as 1.244 (0.688–2.316, 0.492) for anxiety and 1.415 (0.743–2.696, 0.291) for depression levels. The results of both ORs being no statistically different than 1.000 suggested that ‘Intervention’ had no significant impact in moving up or down the levels of anxiety or depression over the RT period.

**Table 5:** Ordered logistic regression (OLR) analysis of three levels of score-based anxiety and depression severity - ‘normal’, ‘borderline’ and ‘abnormal’ after radiotherapy^a,b,c,d^

		Basic OLR analysis			Multivariate OLR analysis		
	Variable	OR	95%CI	*p*-value	OR	95%CI	*p*-value
Anxiety Change	Con.	1.000		Ref.	1.000		Ref.
	Int.	1.384	(0.774–2.474)	0.273	1.244	(0.668–2.316)	0.492
	normal	1.000		Ref.	1.000		Ref.
	borderline	4.825	(2.423–9.611)	<.001	4.178	(1.991–8.764)	0.000
	abnormal	16.350	(5.619–47.573)	<.001	34.295	(9.329–126.078)	<.001
Depression Change	Con.	1.000		Ref.	1.000		Ref.
	Int.	1.611	(0.891–2.910)	0.114	1.415	(0.743–2.696)	0.291
	normal	1.000		Ref.	1.000	Ref.	
	borderline	9.847	(4.909–19.753)	<.001	11.559	(5.381–24.829)	<.001
	abnormal	46.383	(16.756–128.397)	<.001	45.999	(15.546–136.104)	<.001

*OLR* ordered logistic regression, *OR* odd ratio, *95%CI* 95% confidence interval, *Con.* control, *Ref.* reference, *Int.* intervention arm

^a^A few patients were excluded from the basic and multivariable OLR modeling because of their absence of anxiety or depression score changes or TNM stage missing status; covariates in multivariable OLR analysis include age, education, BMI, TNM stage, income level, breast tumor side, and surgery type;

^b^All score tests for the proportional odds assumption were insignificant (*p* > 0.20), which indicated that they met the requirements of using ordered logistic regressions. Basic model consisted of independent variables of study arm and three categories of anxiety or depression severity category at baseline (i.e. before radiotherapy)

^c^ At end, the collapsed groups of ‘Stage III and IV’ and ‘no and elementary school’ had generated the reliable estimates of OR for multivariable OLR analyses on three levels of score-based anxiety and depression categories – ‘normal’, ‘borderline’ and ‘abnormal’; Age and BMI as continuous measurements were incorporated into the multivariable models; statistically, OR refers to the event-occurring odd associated with one-level increase of these ordered categories

^d^Reference groups at OLR analyses are the ‘control’ group of study arm, ‘Stage III and IV’ of TNM stage, ‘college and up’ of education level, ‘right-sided’ of breast tumor side, and ‘BCS’ of surgery type where BCS means the breast conserving surgery

## Discussion

Through extensive analyses, this trial found that both anxiety and depression scores measured in HADS increased over RT course, however, their 3-level category (normal, borderline, abnormal) percents had not significant change. Also, this trial concluded that one comprehensive educational course on BC knowledge with emphasis on teaching stress management skills did not lead to significant changes in either score or category changes of anxiety and depression over the 5–6 weeks of postoperative RT. While its long-term effect still awaits the follow-up data, several aspects are worthy of discussion.

First, this trial studied BC patients admitted for postoperative RT and its conclusion should be interpreted and applied in this setting. Compared to BC patients who do not need postoperative RT, they usually have larger tumors and more metastatic regional lymph nodes in China where the prevalence of patient with early stage BC is still relatively low [46]. Indeed, the ‘abnormal’ rates of having anxiety and depression before RT in this study were as high as 5.3 and 7.1%, respectively. Some prospective studies reported that 3–5% patients at any time point after BC diagnosis met the diagnostic criteria of anxiety and depression under the DSM-IV-TR [20, 21]. In this regard, it is reasonable to believe that BC patients with incoming RT would require more educational courses including RT or longer time for full recovery of distress than other BC populations. In fact, one RCT of 126 BC patients admitted for RT has confirmed the effectiveness of the enhanced knowledge of RT and disease on improving the anxiety and QoL over time from first RT to 3 months after [47]. Therefore, the repetitive and comprhensive courses of BC and its treatments may be more valuable.

Second, the secondary finding of education rather than income level being negatively associated with high anxiety increase needs a careful review. Liteature review seems not to be consistently supportive for this point. Schwarz et al. followed 367 women with breast (*n* = 174) or gynecological (*n* = 193) cancer over 12 months after initial treatments and found the low educational level are at high risk of maintaining high anxiety and depression scores (HADS) over time [48]. However, de Moor studied 487 women with newly diagnosed BC ductal carcinoma in situ (DCIS) and found that instead of education, the financial status was inversely associated with changes in anxiety and depression over 9 months after diagnosis [49]. Chen et al. analyzed 1400 Chinese women with stage 0-IV BC and found that the low income and unmarried marital status were two independent predictors for depression at 18 months post-diagnosis [50]. Given the interacted complex of education, employment, income and marriage, we tended to collectively believe that it would be the socioeconomic status (SES) negatively influence the anxiety and depression symptoms. The low SES often make the adaption process of psycholgical disorders to be longer and harder.

Third, whether and how the Chinese race and culture influenced the trial results is unknown. Lam et al. compared 348 Chinese and 292 German women with breast cancer and found that German reported more anxiety and depression (both *p* < 0.001) and prioritized physical and psychological support [51]. However, You et al. reported that Chinese BC patients (*n* = 97) had higher levels of state anxiety and stronger association of anxiety with QoL than US patients (*n* = 62) after controlling for demographic and medical characteristics [52]. Many researchers often used the social and cultural differences to explain for the racial disparities in depression and anxiety. Lu et al. reported that following the Confucian values, Chinese BC patients often choose to suppress internal emotion, motivation and avoid social cognition and communication of her illness [53]. Also, Chinese cancer patients usually have low health literacy, financial difficulty, advanced stage of disease at initial presentation, and low quality of cancer care compared with US and Europe patients [54, 55]. The authors believe that all of the above could signficantly contribute to development of higher prevalence and severity anxiety and depression in Chinese BC patients.

Like others, this trial has several limitations. Although the Chinese version of HADS has been validated and used in many studies, a few studies still questioned its diagnostic role to assess anxiety and depression [56]. Therefore, the conclusion of statistically insignificant effect of one education course intervention from this study should be treated cautiously. In addtion, this study did not collect the physical activity, exercise and diet data. Recent studies found that their enhancements have positive effects on fatigue and favorable improvement on anxiety and depression over RT period [57, 58]. If the strong BC disease knowledge and management skills can effectively translate into healthy diet, good sleep and regular exercise could not be verified in this study. One study found that the objective measured moderate-to-vigorous physical activity positively associated with health-related QoL indicators including fatigue, depression, and anxiety [59]. One trial of 6-month exercise and hypocaloric healthy eating intervention among 85 BC patients showed a reduction in depressive symptoms [60].

Other limitations of this study included there were no objective measurement data on BC knowledge and skill, the official income, and insurance type being collected. Although both multivariable linear and ordinal logistic regressions were conducted and had reached the same conclusion, the assumed balance between the two randomly-assigned arms could not be confirmed. However, there was no reason to believe that they could reverse the conclusion either. Furthermore, the extended ITT analysis (i.e. included nine “I” arm patients who were excluded from the analyses) did not change the conclusion either. Last, the follow-up measures scheduled at 3 and 5 years in this trial may have new findings.

Finally, it is worthy to note that this study found that these BC patients increased their anxiety and depression scores during the RT period. The RT related complications of poor appetite, gastrointestinal symptoms, slow swelling and pain of breast and arm, and dermatitis could be the causative elements. Further studies of evaluating and managing these causes during RT are warranted.

The clinical implications of this study should exceed the negative finding itself. The moderate high prevalence of anxiety and depression symptoms exists among RT eligible Chinese BC patients and over RT period. A wide range of interrelated risk factors from both disease and non-disease aspects contribute to this dynamic complex overtime. The effective educational and psychological interventions are much needed and tailored to this particular population.

## Conclusions

Compared to the usual care at admission, one additional comprehensive and intensive education course of BC disease and stress management skills did not reduce the anxiety and depression score and severity over the RT period among Chinese patients. Its long-term effect awaits the follow-up data.

## Abbreviations

3DCRT: Three-dimensional conformal radiotherapy
BC: Breast cancer
BCS: Breast conserving surgery
BMI: Body mass index
C: Control
CI: Confidence interval
CTx: Chemotherapy
DSM-IV-TR: The Diagnostic and Statistical Manual of Mental Disorders
ET: Endocrine therapy
HADS: The Hospital Anxiety and Depression Scale
HADS-A: HADS anxiety
HADS-D: HADS depression
I: Intervention
ICF: Informed Consent Form
IMRT: Intensity-modulated radiotherapy
ITT: Intention-to-treatment
MLR: Multivariable linear regression
MOLR: Multinomial ordered logistic regression
MRM: Modified radical mastectomy
OR: Odd Ratio
QoL: Quality of life
RCT: Randomized clinical trial
RT: Radiotherapy
